# Accelerating the discovery of new materials with deep learning

**DOI:** 10.1107/S2052252521013415

**Published:** 2021-12-23

**Authors:** Melanie Vollmar

**Affiliations:** a Diamond Light Source Ltd, Harwell Science and Innovation Campus, Didcot, OX11 0DE, United Kingdom

**Keywords:** high-energy diffraction microscopy, new materials in energy research, deep learning

## Abstract

The use of deep learning and ideas from image recognition to accelerate data analysis and hence discovery of novel materials is described. A particular focus is given to the field of energy research.

Design and development of new materials are crucial to progress and innovation, especially in areas concerned with generation, transmission and use of energy. In recent years, new materials have become even more important, considering the global shift towards renewable energies and sustainability in all areas of the energy sector and the insatiable hunger for energy consumption (Ma *et al.*, 2021 ▸; Kordas, 2017 ▸).

Commonly, new materials are proposed based on intuition, past experience and studying empirical results. However, pushing the boundaries to explore as yet under-studied areas of potential materials is not routinely done. This is due to a combination of factors. On one hand, there is the difficulty of breaking out of established design strategies to identify novel materials, and on the other hand, there is the challenge of accelerating existing imaging techniques and the software required to analyze the acquired experimental data for these new materials. Taking a lead from the developments in machine learning and artificial intelligence, the first issue has been addressed by using a number of different algorithms to design novel materials as is reviewed in Liu *et al.* (2021 ▸). To investigate newly designed materials and to verify that the produced sample is consistent with theoretical assumptions, high-energy X-ray characterization methods such as high-energy diffraction microscopy (HEDM) (Park *et al.*, 2017 ▸) have become routinely available over recent years. HEDM is based on three-dimensional X-ray diffraction which provides a non-destructive way to analyze the internal grain structure of materials (Poulsen *et al.*, 2001 ▸; Poulsen, 2012 ▸). Use of diffraction and tomographic imaging elucidates the inner granular structure of materials down to the micrometre level. Nevertheless, the analysis of such data remains computationally expensive. Liu *et al.* (2022 ▸) use deep learning to address the second shortcoming of new materials development, the lack of fast data analysis.

While rotation imaging of a sample can be completed within minutes, for example at a synchrotron facility, the analysis of the data is substantially slower. In particular, finding Bragg peaks and determining their precise peak location, as well as reconstructing the material grain structure based on this information, are very time consuming. A Voigt profile in 2D or 3D needs to be fitted to determine the peak location, which is computationally expensive, hence, a pseudo-Voigt is usually used for approximation. After identifying the center-of-mass of the Bragg peaks, the individual grains in the material are reconstructed (Sharma *et al.*, 2012*a* ▸,*b* ▸). Even so, depending on the nature of the material under investigation and computational resources available, it can take up to several weeks to determine the precise peak positions and reconstruct the grain structure. As such, the slow processing and analysis of HEDM data in material sciences hampers a routine trial-and-error assessment of newly designed materials and dramatically reduces the speed of new discoveries.

In the current issue of **IUCrJ** , Liu *et al.* (2022 ▸) have developed a deep neural network based system, *BraggNN*, to precisely locate Bragg peaks in HEDM images. In their machine learning-based model (Fig. 1 ▸), Liu *et al.* (2022 ▸) build on the established and well documented work around convolutional neural networks (Fukushima, 1980 ▸) in image recognition. The aim for this system is to provide a near real-time data analysis and feedback application – greatly reducing the time needed for data analysis compared with standard methods (Abeykoon *et al.*, 2019 ▸). Its sole purpose is to rapidly identify the center-of-mass of a Bragg peak to sub-pixel precision. Fig. 2 ▸ compares three methods, near-field HEDM, pseudo-Voigt far-field HEDM and *BraggNN*, and how well each performed in identifying the precise location of the Bragg peaks, allowing for the reconstruction of the grains in the sample material. As can be seen, applying the deep learning algorithm *BraggNN* enables the correct reconstruction of all but a few grains, usually at the edge of the sample, while at the same time greatly reducing the analysis time. Compared with optimized pseudo-Voigt fitting (Sharma *et al.*, 2012*a* ▸) a 57× reduction in computation time was achieved on a CPU system and a 350× reduction when GPUs were accessed. Even a good graphics card GPU allowed for a 250× reduction in computational time for center-of-mass detection for the Bragg peaks compared with standard pseudo-Voigt fitting. So, even groups with limited computational resources can benefit greatly from this new algorithm.

*BraggNN* is one step towards on-the-fly data analysis for the field of material sciences. If the application described here proves as efficient and robust as it was for the sample used for development, then this new approach to HEDM data analysis offers a significant step forward to assess new materials more quickly and increase the turn-over rate from theoretical conception to practical application. ▸

## Figures and Tables

**Figure 1:**
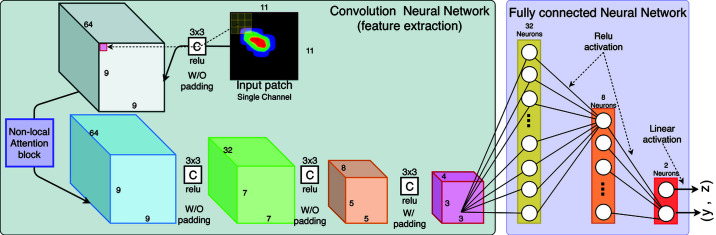
Neural network architecture as published by Liu *et al.* (2022 ▸). The image shows the details for the convolutional part of the network used for feature extraction and the fully connected part used for inference.

**Figure 2:**
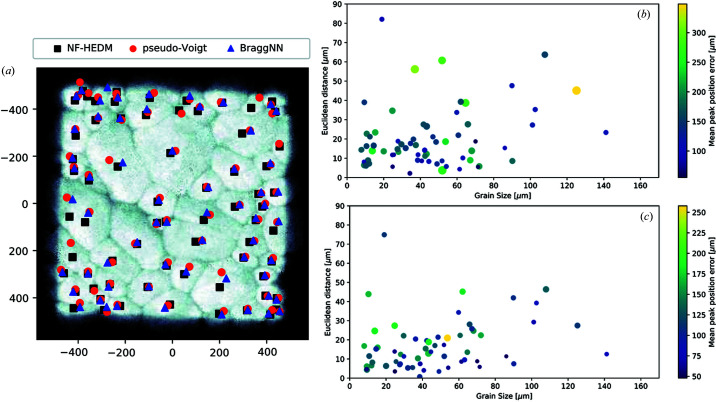
Grain identification by three different algorithms. Comparison between near-field HEDM, pseudo-Voigt far-field HEDM and *BraggNN* for the test material used by Liu *et al.* (2022 ▸). (*a*) Gives the grain position in the material for near-field HEDM (black squares), pseudo-Voigt far-field HEDM (red circles) and *BraggNN* (blue triangles). (*b*)–(*c*) Represent the differences in the grain positions between near-field HEDM (*b*), pseudo-Voigt far-field HEDM (*c*) and *BraggNN* as determined Liu *et al.* (2022 ▸).
